# Feasibility Studies of Nebulized SARS-CoV-2 Neutralizing Antibody in Mice and Cynomolgus Monkeys

**DOI:** 10.1007/s11095-022-03340-9

**Published:** 2022-07-26

**Authors:** Jilei Jia, Zhaojuan Yin, Xiao Zhang, Huimin Li, Dan Meng, Qianqian Liu, Hongfang Wang, Meng Han, Shixiang Suo, Yan Liu, Ping Hu, Chunyun Sun, Jing Li, Liangzhi Xie

**Affiliations:** 1 Sinocelltech Ltd., No.31 Kechuang 7th Street, Beijing, 100176 BDA China; 2 Beijing Engineering Research Center of Protein and Antibody, Sinocelltech Ltd, No.31 Kechuang 7th Street, Beijing, 100176 BDA China; 3 Cell Culture Engineering Center, Chinese Academy of Medical Sciences & Peking Union Medical College, Beijing, 100005 China

**Keywords:** aerosol, inhalation, neutralizing antibody, pharmacokinetics, SARS-CoV-2

## Abstract

**Purpose:**

Neutralizing antibodies, administrated through intravenous infusion, have shown to be highly efficacious in treating mild and moderate COVID-19 caused by SARS-CoV-2 infection in the lung. However, antibodies do not transport across the plasma-lung barrier efficiently, and up to 100 mg/kg dose was used in human causing significant supply and cost burdens. This study was to explore the feasibility of nebulized antibodies inhalation delivery as an alternative route.

**Methods:**

HB27, a potent RBD-specific humanized monoclonal antibody (Zhu et al. in National Sci Rev. 8:nwaa297, 2020), showed excellent protection against SARS-CoV-2 in animal model and good safety profile in clinical studies. The pharmacokinetics and preliminary safety of HB27 administrated through the respiratory tract were studied in mice and cynomolgus monkeys here.

**Results:**

At a single 5 mg/kg dose, the peak HB27 concentration in mice pulmonary epithelial lining fluid (ELF) reached 857.8 μg/mL, 670-fold higher than the PRNT_90_ value of 1.28 μg/mL, and maintained above PRNT_90_ over 240 h. In contrast, when administrated by intravenous injection at a 5 mg/kg dose, the antibody concentrations in mice ELF were below PRNT_90_ value throughout, and were about 50-fold lower than that in the serum. In cynomolgus monkeys administrated with a single dose through inhalation, the antibody concentration in ELF remained high within 3 days. No drug-related safety concerns were observed in the studies.

**Conclusions:**

The study demonstrated that nebulized neutralizing antibody delivery though inhalation could be a more efficient and efficacious alternative approach for treating COVID-19 and other respiratory infectious diseases, and warrants further evaluation in clinical studies.

**Supplementary Information:**

The online version contains supplementary material available at 10.1007/s11095-022-03340-9.

## Introduction

Coronavirus disease 2019 (COVID-19) is still going on with the emergence of SARS-CoV-2 and its variants, especially the Omicron variant strain, which has worsened the global anti-epidemic situation. According to World Health Organization (WHO) statistics [2], as of April 2022, there have been more than 0.5 billion confirmed cases of COVID-19 worldwide, including over 6 million deaths.

Several neutralizing antibody or antibody combination therapies have been approved with Emergency Use Authorizations (EUA) for COVID-19 treatment or pre-exposure prophylaxis. These antibodies are administered by intravenous infusion or intramuscular injection [3–12]. HB27 is a high affinity (K_D_ 67 pM) and potent neutralizing antibody against SARS-CoV-2 with a molecular weight of 146 kDa [1]. HB27 neutralizes SARS-CoV-2 PsV with an IC_50_ of 0.04 nM and an authentic SARS-CoV-2 strain with a PRNT_50_ value of 0.22 nM. Its antiviral potency was further demonstrated by > 1000-fold reduction in lung viral load in mouse intranasal viral challenge models, after a single dose of 20 mg/kg of HB27 intravenous administration. In non-human primates, the average half-life was 10.0 ± 2.2 days and no obvious adverse events were observed when HB27 was administered by intravenous injection in a single dose of 150 and 500 mg/kg [1]. A phase I clinical study in healthy participants in China showed good safety and tolerability [13]. Subsequently, it has been approved by the US FDA and regulatory agencies in other countries to conduct phase 2/3 clinical trials (NCT04644185) in outpatients and hospitalized COVID-19 patients.

The monoclonal antibody therapies have shown excellent efficacies and safety profiles in treating mild and moderate COVID-19 patients. However, SARS-CoV-2 tends to be concentrated in respiratory system such as lungs, with very little in the bloodstream [14, 15]. A therapeutic antibody administered by intravenous injection needs to cross the plasma-lung barrier to exert its efficacy. Large biomolecules such as monoclonal antibodies don’t transport through the plasma-lung barrier efficiently [16]. As a result, dosages of up to 100 mg/kg antibodies were used to treat COVID-19 patients in clinical trials [17, 18], which put significant pressure on treatment cost and antibody production, thus limiting the widespread use of mAbs in developing countries [19].

Respiratory administration (aerosol inhalation, for instance), is an efficient drug delivery alternative with lung as the main target organ. Compared with intravenous injection, inhalation has advantages such as convenient application, rapid onset, high local drug concentration, less dosage and low systemic exposure so as to reduce potential side effects. This will undoubtedly provide a new strategy for the treatment of COVID-19 pulmonary infection. Not surprisingly, therapeutic candidates with intrinsic short pharmacokinetics in the blood, such as small molecule inhibitors [20], fragmented antibodies [21, 22], and nanobodies [23–25] have been developed for aerosol inhalation administration to treat COVID-19. On the other hand, there are few studies on atomization inhalation administration of full-length antibodies with much longer T_1/2_ in the blood, presumably due to developmental challenges and concerns of antibody aggregation or degradation after atomization [26–31]. In fact, few clinical study has been conducted to date with inhaled antibodies. In this study, pharmacokinetic characteristics of nebulized HB27 inhalation were studied in mice and cynomolgus monkeys.

## Materials and Methods

### The Recombinant Neutralizing Antibody against SARS-CoV-2

HB27 is an IgG1 monoclonal antibody with a molecular weight of 146 KDa, which is developed with phage-display scFv library and antibody humanization technology. The Fc region is modified with ‘LALA’ double mutation (Leu234Ala together with Leu235Ala) to reduce antibody-dependent enhancement effect without affecting the binding ability with FcRn. It was produced in Chinese hamster ovary (CHO) cells, purified with multi-steps of chromatography, and tested to meet quality control standards for clinical studies.

### Bioactivity and Characterization

*In vitro* neutralization activity of HB27 was evaluated with pseudovirus (Wuhan-Hu-1 strain) before administration. 50 μL serial diluted antibody was mixed with 50 μL 100TCID_50_ pseudovirus in 96-well plate and incubated at 37℃ in 5% CO_2_ incubator for 1 h. Then, 3 × 10^4^ 293FT-ACE2 cells were inoculated at 100 μL per well for another 20 h. After incubation, the culture supernatant was removed and 1 × Passive lysis buffer was added at 50 μL/well to lyse the cells. The cell lysate was transferred into 96-well white bottom plate at 40 μL/well with 40 μL/well luciferase substrate added. Luminescence value (RLU) was detected by Microplate Luminometer to calculate the neutralization. Neutralization (%) = (Positive Control RLUs — Sample RLUs) / (Positive Control RLUs — Negative Control RLUs) × 100%. The dose–effect curve was analyzed and drawn by GraphPad Prism software, the abscissa was the logarithm of the sample concentration, and the ordinate was the neutralization. Furthermore, the stability and binding activity of nebulized HB27 were briefly analyzed with size exclusion high-performance liquid chromatography (SEC-HPLC) and ELISA.

### Intratracheal Aerosol Administration and BALF Collection in Mice

**Fig. 1:**
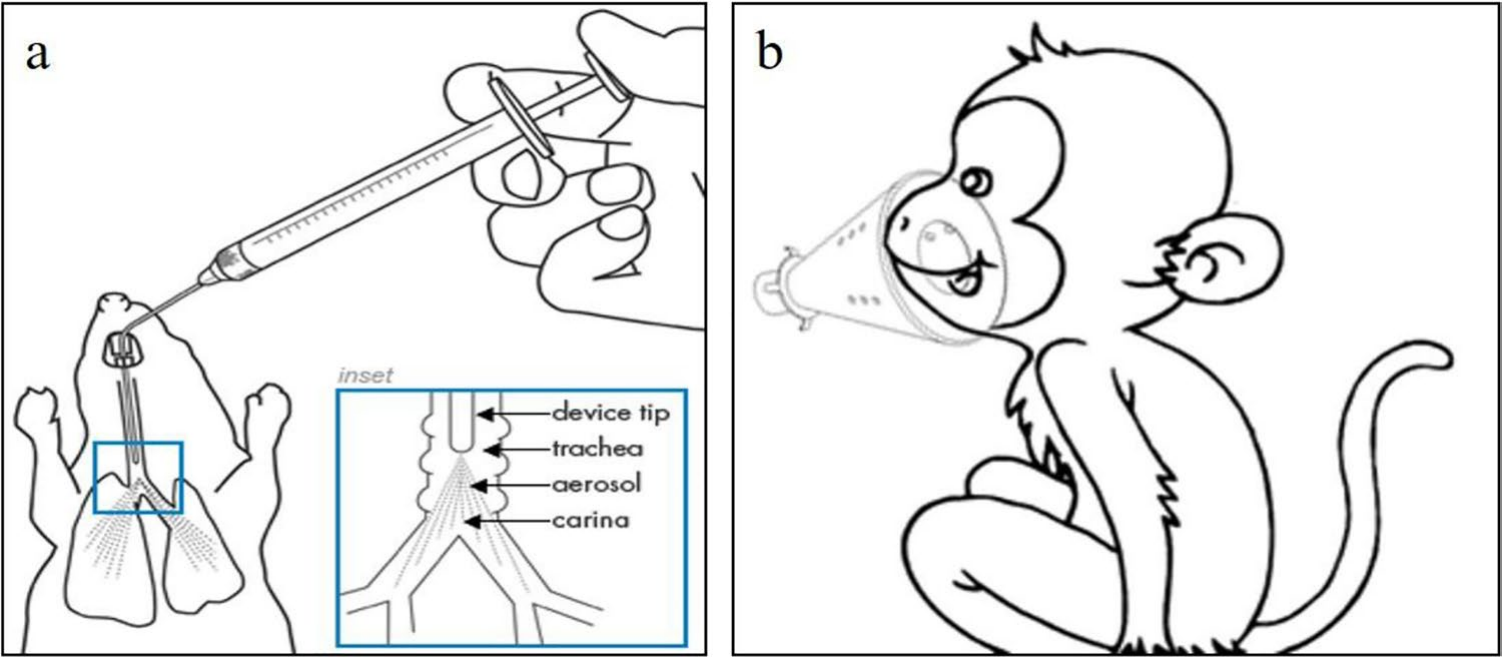
Schematic diagram of administration operation. (**a**) Intratracheal aerosol nebulization in mice; (**b**) Facial inhalation using Jet NE-C28 Nebulizer for Cynomolgus monkeys.

Mice were anesthetized by intraperitoneal injection of 50 mg/kg 1% pentobarbital Na (Merck). The animal was fixed on an operating table with an angle of about 20° from the horizontal plane [32, 33]. A miniature liquid aerosolizer needle (HRH-MAG4, Beijing HuiRongHe Technology Co., Ltd) was inserted into the trachea exhalation and inhalation. The antibody was nebulized into the lungs in one shot (Fig. 1a).

After the mice were anesthetized, blood samples were collected from the orbital venous plexus for serum analysis. Broncho-alveolar lavages fluid (BALF) was collected using the tracheostomy intubation method, and the lavage procedure was performed immediately after blood collection [34]. A small incision in the trachea was made to aspirate 0.5 mL PBS (18073001, Origene Wuxi Biotechnology Co., Ltd.) with a 23G flat-tipped needle (Zibo Furui Medical Instrument Co., Ltd.) for lavage. The lung lavage fluids and serum samples were stored at -40°C.

### Aerosol Inhalation Administration and BALF Collection in Cynomolgus Monkeys

The jet NE-C28 nebulizer (Omron (Dalian) Co., Ltd.) has a breathing mask for drug delivery [35, 36]. All animals were awake and breathing spontaneously during aerosol inhalation administration (Fig. 1b). BALF collection in cynomolgus monkeys was performed after euthanasia of the animals. The left and right lungs were treated with lavage for three times each with 10 mL PBS solution to ensure adequate lavage, and BALF samples were frozen at -80°C in 50 mL EP tubes for antibody concentration and cytokines detection.

### Detection and Analysis of Antibody Concentrations in Serum and BALF/ELF

The antibody concentrations in serum and BALF were detected by indirect ELISA method. ELISA plates were coated with 0.5 μg/mL RBD protein as a capture reagent and incubated at 2–8°C overnight. Standard samples, quality controls and test samples were added to the coated ELISA plate, and incubated for 2 h at room temperature (RT), and then washed 3 times. 15 ng/mL goat anti-human IgG-Fc Secondary Antibody (HRP) secondary antibody (SSA001, Sino Biological Inc.) was added and incubated in the dark for 1 h at RT, and washed again. Chromogenic solution was added and incubated in the dark for approximately 10–13 min at RT. Finally, 2 mol/L H_2_SO_4_ was added to stop the color reaction, and the OD values were read at a wavelength of 450 nm. Pharmacokinetic (PK) parameters were calculated using non-compartmental model (NCA) in Phoenix-WinNonlin 8.1 software.

Antibody concentration in epithelial lining fluid (C_ELF_) was calculated from the antibody concentration measured in the BALF (C_BALF_) and corrected via urea nitrogen concentration measurements. Since urea nitrogen can diffuse freely across the plasma-lung barrier, the urea nitrogen concentration in ELF (U_ELF_) should equal that in serum (U_serum_), hence the ratio of U_serum_/U_BALF_ represents the dilution factor introduced through the BALF acquisition process and can be used to correct the actual antibody concentration in the ELF. The urea nitrogen content in serum and BALF was detected using a urea detection kit (MAK006, Sigma), and the OD value was read at 450 nm on a Bio Tek Epoch photometer (EPOCH2, BioTek). The formula for calculating C_ELF_ is:

$${\mathrm C}_{\mathrm{ELF}}\;(\mathrm\mu\mathrm g/\mathrm{mL})={\mathrm C}_{\mathrm{BALF}}\;(\mathrm\mu\mathrm g/\mathrm{mL})\times{\mathrm U}_{\mathrm{serum}}\;(\mathrm{nmol}/\mathrm{mL})/{\mathrm U}_{\mathrm{BALF}}\;(\mathrm{nmol}/\mathrm{mL})$$

Or in mice:

$${\mathrm C}_{\mathrm{ELF}}\;(\mathrm\mu\mathrm g/\mathrm{mL})={\mathrm C}_{\mathrm{BALF}}\;(\mathrm\mu\mathrm g/\mathrm{mL})\times21.2.$$

From the measurements in the first study in mice, the mean of U_serum_/U_BALF_ was calculated as 21.2. The BALF was collected by a standardized process with almost the same volume for each mouse, so the U_serum_/U_BALF_ is basically a constant. Hence, the C_ELF_ in mice was calculated by multiplying C_BALF_ by 21.2 in the subsequent studies.

### Single-dose Intravenous Injection of HB27 in C57BL/6 Mice

C57BL/6 mice (6–8 weeks old, 18–24 g) were supplied by Vital River Laboratories (Beijing, China). 60 mice were randomly assigned to 5 mg/kg and 50 mg/kg dose groups. All mice were administered by a single intravenous injection with the same volume of 10 mL/kg. Six mice from each group were euthanized to collect serum and BALF samples at each of designated time points (1 h and 6 h in 5 mg/kg dose group, 24 h, 48 h, 72 h and 168 h in both groups) after dosing.

### Intratracheal Aerosol Administration in C57BL/6 and BALB/c Mice

To confirm whether there are differences on antibody distribution between animal strains after intratracheal aerosol administration of HB27, 8 C57BL/6 mice and 6 BALB/c mice were studied. All mice were supplied by Vital River Laboratories (Beijing, China). The mice were administered with a single intratracheal inhalation of 5 mg/kg HB27 with 100 μL per animal. Serum and BALF samples were collected for comparison at 24 h after dosing.

HB27 was administered by intratracheal inhalation at a single dose of 5 mg/kg with 100 μL per animal for the PK profile determination in 48 BALB/c mice. Six animals (3/sex/time point) were euthanized to collect serum and BALF samples at each of the designated time points (6 h, 24 h, 48 h, 72 h, 96 h, 168 h, 240 h and 336 h) after dosing, respectively.

A repeat-dose PK study was also conducted in 20 BALB/c mice. The mice from each group (5/sex/group) were treated with vehicle or 5 mg/kg HB27 by intratracheal atomization once daily for 5 days, respectively. The inhalation volume was 100 μL per animal for the first four dosing, and 50 μL per animal for the last dosing. Serum and BALF samples were obtained at 24 h after the last dosing.

### Single Aerosol Inhalation to Cynomolgus Monkeys

A total of 10 cynomolgus monkeys, 4–7 years old, half female and half male, were used in this study. Before the experiment starts, a mock nebulization process was conducted in one female cynomolgus monkey to determine air flow rate (mL/min, AR) and average nebulization rate (mL/min, ANR) of the nebulizers. The ANR of each nebulizer was measured for 4 times in 4 periods of time (4, 6, 4 and 6 min). And the mean ANR was calculated from 4 measurements to be used in the subsequent calculation. The ANR was calculated as follows:

$$\mathrm{ANR}\;(\mathrm{mL}/\min)=\mathrm{Dosing}\;\mathrm{Volume}\;(\mathrm{mL})/\mathrm{Inhalation}\;\mathrm{exposure}\;\mathrm{time}\;(\mathrm T,\;\min).$$

The aerosol antibody concentration (mg/mL, C_A_) can be calculated from the AR, the mean ANR and formulation concentrations of HB27 (C_P_) as follows:

$${\mathrm C}_{\mathrm A}(\mathrm{mg}/\mathrm{mL})=\mathrm{ANR}\;(\mathrm{mL}/\min)\times{\mathrm C}_{\mathrm P}\;(\mathrm{mg}/\mathrm{mL})/\mathrm{AR}\;(\mathrm{mL}/\min).$$

Respiratory minute volume (mL/min, RMV) should be measured prior to dosing by Emka PACK 4G non-invasive animal physiological signal telemetry system (A8826E, Emka Technologies Inc.). The other 9 monkeys were exposed to 10 mg/kg HB27 antibody with single facial aerosol inhalation using 3 jet-type NE-C28 nebulizers. The achieved dose of HB27 antibody in different positions of the respiratory tract after being inhaled by the nebulizers is estimated according to the method described by Vonarburg [29].

$$\begin{array}{c}\mathrm{Total}\;\mathrm{Delivered}\;\mathrm{Dose}\;(\mathrm{mg}/\mathrm{kg},\;\mathrm{TDD})=({\mathrm C}_{\mathrm A}\times\mathrm{RMV}\times\mathrm T)/\mathrm{BW},\\\mathrm{Delivered}\;\mathrm{Lung}\;\mathrm{Dose}\;(\mathrm{mg}/\mathrm{kg},\;\mathrm{DLD})=({\mathrm C}_{\mathrm A}\times\mathrm{RMV}\times\mathrm T)/\mathrm{BW}\times\mathrm{LDF},\\\mathrm{Lung}\;\mathrm{Dose}\;(\mathrm{mg}/\mathrm g\;\mathrm{lung}\;\mathrm{tissue},\;\mathrm{LD})=({\mathrm C}_{\mathrm A}\times\mathrm{RMV}\times\mathrm T)/\mathrm{LW}\times\mathrm{LDF},\end{array}$$

where C_A_ is antibody concentration in aerosol (mg/mL), RMV is respiratory minute volume (mL/min), T is inhalation exposure time (min), BW is body weight (kg), LDF is lung deposition factor (taken as 10% of inhaled dose), and LW is lung weight (g).

Three monkeys were euthanized per time point to collect BALF at 24 h (1 female and 2 males), 48 h (2 females and 1 male) and 72 h (1 female and 2 males) after administration. Serum samples were collected prior to dosing and at 1 h, 6 h, 24 h, 48 h and 72 h after administration. The antibody concentrations and urea nitrogen contents in BALF and serum samples were detected by indirect ELISA and urea nitrogen detection kit, respectively. In this study, we also use flow cytometry to determine concentrations of cytokines, including TNF-α, IFN-γ, IL-2, IL-6, IL-13, IL-5, IL-4 and IL-10, in BALF. The measured antibody and cytokine concentrations in the BALF was converted to concentrations in the ELF using the conversion factor of U_serum_/U_BALF_. The concentration of HB27, urea nitrogen and cytokines level in the left and right lungs were tested separately. Concerned tissues, including turbinate, paranasal sinus, pharynx, larynx, trachea, main bronchus and lung tissues, were evaluated for macroscopic and microscopic examinations.

### Statistical Analysis

SPSS (16.0) and GraphPad Prism (8.0) were used for statistical analyses and graphs, respectively. All data were expressed as mean ± standard deviation (Mean ± SD). Statistical comparisons were performed using one-way ANOVA test and statistical significance was defined as a P value < 0.05.

## Results

### HB27 Bioactivity and Characterization of Nebulized Antibody

**Fig. 2:**
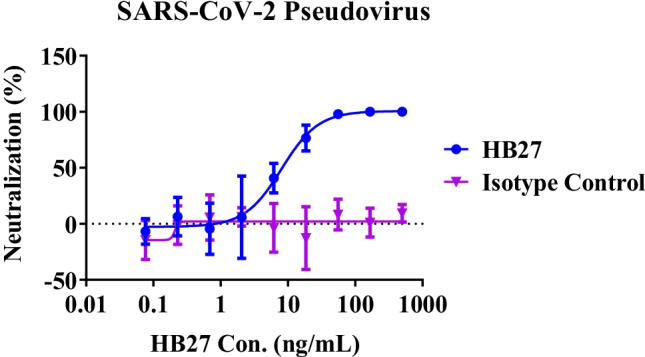
Neutralizing activity of HB27 antibody against SARS-CoV-2 pseudovirus *in vitro* (Mean ± SD).

**Fig. 3:**
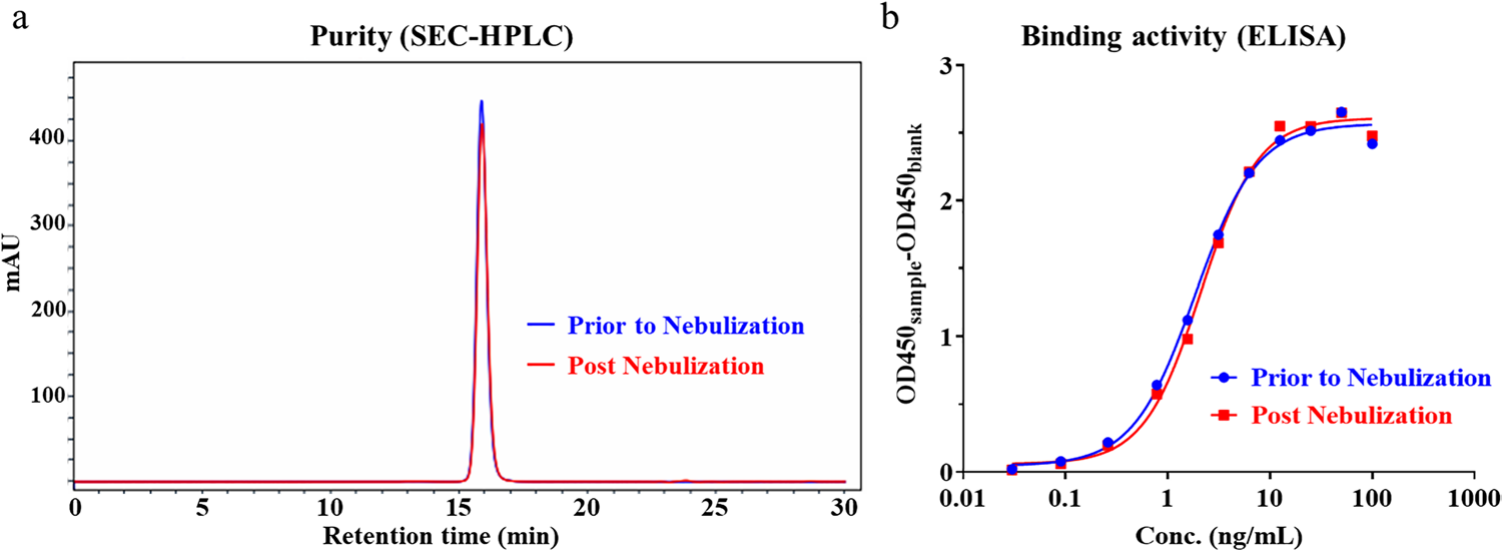
Assessment of HB27 stability and binding activity upon nebulization. (**a**) The purities of the samples prior to nebulization (blue chromatogram) and post nebulization (red chromatogram) were analyzed with SEC-HPLC; (**b**) The RBD-binding activities were detected by ELISA.

The IC_50_ and IC_90_ values of HB27 antibody were measured in a pseudovirus assay to be 8.0 ng/mL and 32.0 ng/mL, respectively (Fig. 2), which is consistent with bioactivity data published [1]. HB27’s broad cross-neutralization activities against multiple SARS-CoV-2 variants have been reported previously [37] and the data are summarized in Supplementary Figure S1. SEC-HPLC and ELISA were used to monitor antibody quality changes after nebulization (Fig. 3 and Table S1). No significant changes in antibody monomer, aggregate, and fragment percentages were detected after nebulization. Bioactivities measured by RBD-binding ELISA were also unchanged from the nebulization process.

### C_ELF_ is About 50-fold Lower than C_serum_ via Intravenous Injection

Since SARS-CoV-2 virus replicates primarily in the respiratory tract, viral load in serum is often under detection limit [38–40]. Hence, it is more important to measure antibody concentration profile in the lung tissue where the battle ground is located. However, measuring antibody concentration in lung tissue is not feasible in human, therefore it is difficult to determine the optimal intravenous dose with serum PK data.

**Fig. 4:**
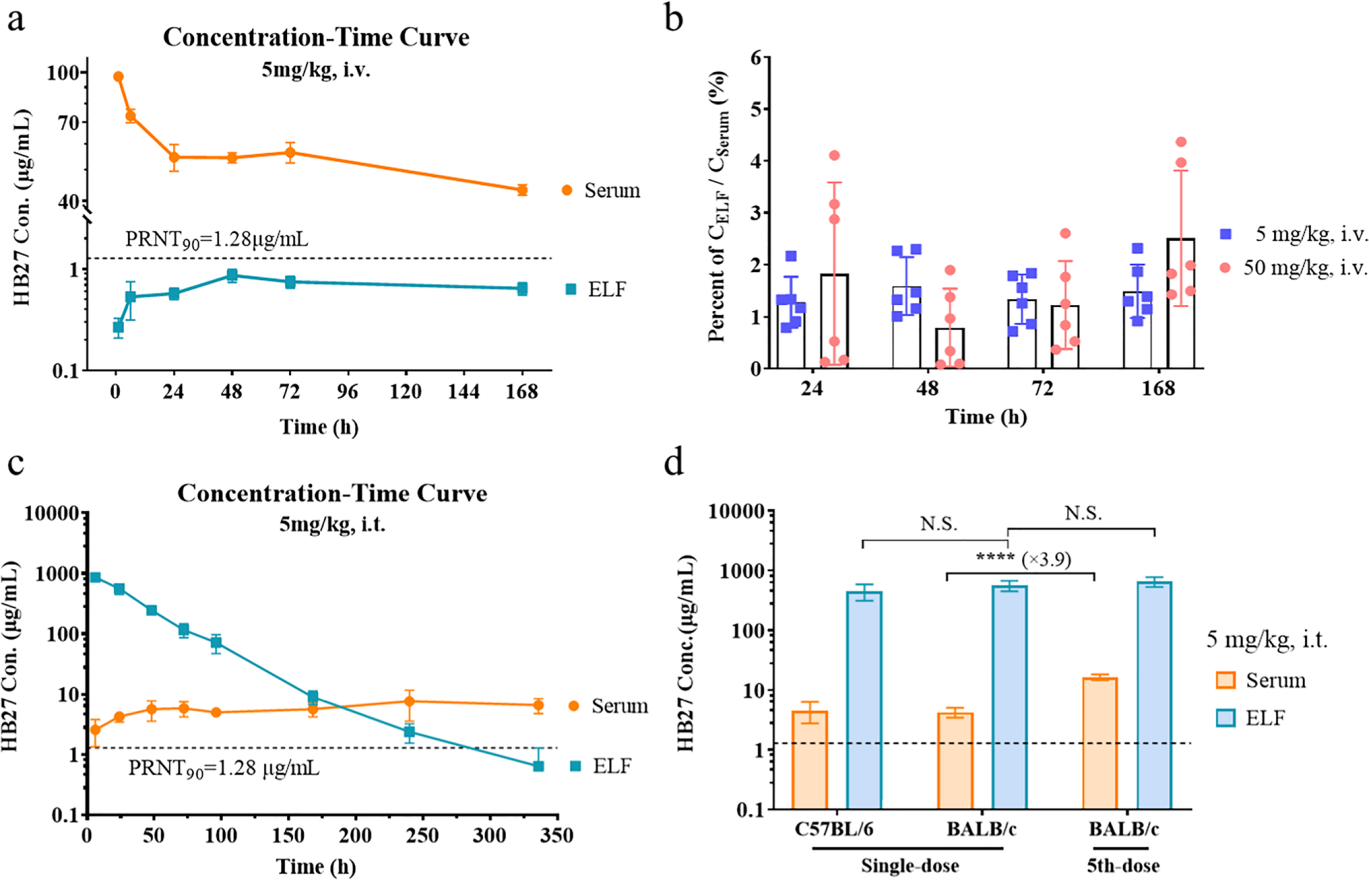
HB27 antibody concentrations in serum and ELF after intravenous administration (i.v.) or intratracheal nebulization (i.t.) in mice (n = 6). (**a**) Concentration–time profiles in serum and ELF were compared after intravenous administration of 5 mg/kg HB27 antibody in C57BL/6 mice (Mean ± SD). Dashed lines represent *in vitro* neutralization activity of HB27 against authentic SARS-CoV-2 by plaque reduction neutralization test (PRNT) in Vero cells [1]. (**b**) The percentages of C_ELF_ / C_serum_ in different time points are shown as Mean ± SD. (**c**) Concentration–time profiles in serum and ELF were compared after a single dose of 5 mg/kg nebulized HB27 antibody to BALB/c mice. (**d**) Antibody concentrations (Mean ± SD) in serum and ELF were detected at 24 h after dosing with a single dose of 5 mg/kg HB27 to C57BL/6 mice and BALB/c mice or 5 repeated doses (once daily) in BALB/c mice (N.S., not significant; ∗∗∗∗ , P < 0.0001).

In our study, we measured the antibody concentration profiles in serum and ELF in mice injected with one single intravenous dose of HB27 antibody at 5 and 50 mg/kg. As shown in Fig. 4a and S2, antibody concentrations in serum and ELF were dose-related, but the concentrations in ELF were significantly lower than those in serum between 24–168 h at both dose levels. Compared with the neutralization activity against authentic SARS-CoV-2 by plaque reduction neutralization test (PRNT) in Vero cells [1], the HB27 antibody concentrations in ELF at 5 mg/kg dose were higher than the PRNT_50_ (32 ng/mL), but lower than the PRNT_90_ (1.28 μg/mL), suggesting incomplete inhibition of viral replication in the lung. The antibody concentrations in ELF at 50 mg/kg were several-fold higher than the PRNT_90_ throughout the study course.

The C_ELF_/C_serum_ ratios represent the antibody delivery efficiencies to the lung tissue through intravenous administration. As expected, due to low efficiency in crossing the plasma-lung barrier, the antibody concentrations in ELF were about 50-fold lower than those in the serum at all time points measured (Fig. 4b). The ratios were almost the same at the two dosages tested.

### Single-inhaled Pharmacokinetics of Nebulized HB27 in C57BL/6 and BALB/c Mice

The PK characteristics of aerosolized antibody delivery via respiratory tract were studied in two mouse models with a single inhalation dose. After administration of 5 mg/kg of HB27 antibody to mice by intratracheal inhalation, serum antibody concentrations at 24 h were 4.54 and 4.24 μg/mL in C57BL/6 and BALB/c mice, respectively. The antibody concentrations in ELF were 448.70 and 560.42 μg/mL in C57BL/6 and BALB/c mice, respectively, approximately 100-fold higher than the serum antibody concentrations. No significant differences between the two mouse strains (P > 0.05) were observed (Fig. 4d).

The single-dose PK study was carried in BALB/c mice with nebulized HB27 antibody at a 5 mg/kg dose. The antibody concentration in ELF at 6 h reached 857.82 μg/mL and gradually decreased over time, but maintained above the PRNT_90_ over 240 h (Fig. 4c and Table S2), which represented potent and lasting efficacy against SARS-CoV-2. On the other hand, serum antibody concentrations increased slightly after intratracheal nebulization and remained a low level within 2 weeks. But they were continuously higher than PRNT_90_, demonstrating the sufficient ability to eliminate systemic viruses. The PK parameters in ELF were calculated and shown in Table 1. T_1/2_ was 44.4 h, which is consistent with that in the literature [29].

**Table 1:** Pharmacokinetic parameters in ELF after single inhalation of 5 mg/kg nebulized HB27 antibody to BALB/c mice (6–336 h)

Parameter	T_1/2_	T_max_	C_max_	AUC_last_	AUC_INF_	Vz	Cl	MRT_last_
	(h)	(h)	(μg/mL)	(h*μg/mL)	(h*μg/mL)	(mL/kg)	(mL/h/kg)	(h)
Value	44.4	6.0	857.8	35,054.5	35,095.2	9.1	0.14	37.8

### Repeat-dose Study of Nebulized HB27 Antibody in Mice

In another repeat-dose study, 5 mg/kg nebulized HB27 antibody was administered to BALB/c mice by intratracheal nebulization once daily for 5 doses in total. The antibody concentrations in serum and ELF at 24 h after the last dose were 16.58 ± 1.81 and 653.34 ± 123.56 μg/mL. As expected, HB27 concentrations in the ELF were about 40-fold higher than concentrations in serum. Comparing with the single-dose study, the antibody in ELF showed no significant accumulation, while the serum antibody concentration accumulated 3.9-fold after 5 repeated doses (Fig. 4d). Nonetheless, the serum antibody concentration was still lower than that in a single intravenous dose.

The dosing volume was 100 μL per mouse at the start of this study, but some mice developed shortness of breath after the 3rd or 4th dosing, presumably due to small lung capacity of mice. When the dosing volume was reduced to 50 μL, the above symptoms disappeared. It is more suitable and safer with the volume of equal to or below 0. 05 mL for intratracheal aerosol administration in mice. The euthanized mice after the last dose of HB27 antibody showed no drug related abnormities in the macroscopic examination, compared with those in the vehicle group.

### Lung PK and Safety Profile of Inhaled HB27 Antibody in Monkeys

Based on the positive results in mice, the aerosol inhalation of HB27 antibody was further explored in cynomolgus monkeys. The mean average nebulization rates (ANRs) of the three nebulizers used in this study were measured to be 0.23, 0.19, and 0.24 mL/min with a mock nebulization process, and the aerosol antibody concentrations (C_A_s) were measured to be 0.00045, 0.00041, and 0.00039 mg/mL, respectively (Table S3). The respiratory minute volume (RMV) of each monkey was measured prior to nebulization by Emka system. The cynomolgus monkeys were randomly assigned into three groups, euthanized at 24 h, 48 h and 72 h after facial inhalation of nebulized HB27 antibody, respectively. The estimated Total Delivered Doses (TDDs) for three groups were 3.92 ± 0.31, 5.22 ± 4.68 and 4.78 ± 1.77 mg/kg and the average TDD among all monkeys was 4.64 mg/kg. The TDDs were approximately half of the theoretical dose of the liquid formulation (10 mg/kg), due to losses in the nebulization process. In consideration of the lung deposition factor in NHPs (about 10%) [29], the average Delivered Lung Doses (DLDs) were only 0.39, 0.52 and 0.48 mg/kg, and Lung Doses (LDs) were 0.08, 0.10 and 0.10 mg/g lung tissue, respectively (Table 2).

**Table 2:** Adjusted dose of a single aerosol inhalation of theoretical 10 mg/kg HB27 liquid formulation in cynomolgus monkeys

Time	Animal ID	RMV (mL/min)	BW (kg)	TDD (mg/kg)	DLD (mg/kg)	LW (g)	LD (mg/g)
24 h	1#	2112.5	2.8	4.25	0.42	14.783	0.08
	5#	2527.6	5.0	3.86	0.39	26.157	0.07
	6#	1890.6	4.6	3.64	0.36	20.825	0.08
	**Mean**	**2176.9**	**4.1**	**3.92**	**0.39**	**20.588**	**0.08**
	SD	323.3	1.2	0.31	0.03	5.691	0.01
48 h	2#	1221.7	2.9	2.76	0.28	20.271	0.04
	3#	905.4	3.1	2.28	0.23	17.214	0.04
	7#	4710	4.6	10.61	1.06	23.471	0.21
	**Mean**	**2279.0**	**3.5**	**5.22**	**0.52**	**20.319**	**0.10**
	SD	2111.2	0.9	4.68	0.47	3.129	0.10
72 h	4#	1810.3	3.2	2.74	0.27	16.313	0.05
	8#	3908.8	4.4	5.78	0.58	23.001	0.11
	9#	3628.7	5.5	5.83	0.58	24.397	0.13
	**Mean**	**3115.9**	**4.4**	**4.78**	**0.48**	**21.237**	**0.10**
	SD	1139.4	1.2	1.77	0.18	4.321	0.04

**Fig. 5:**
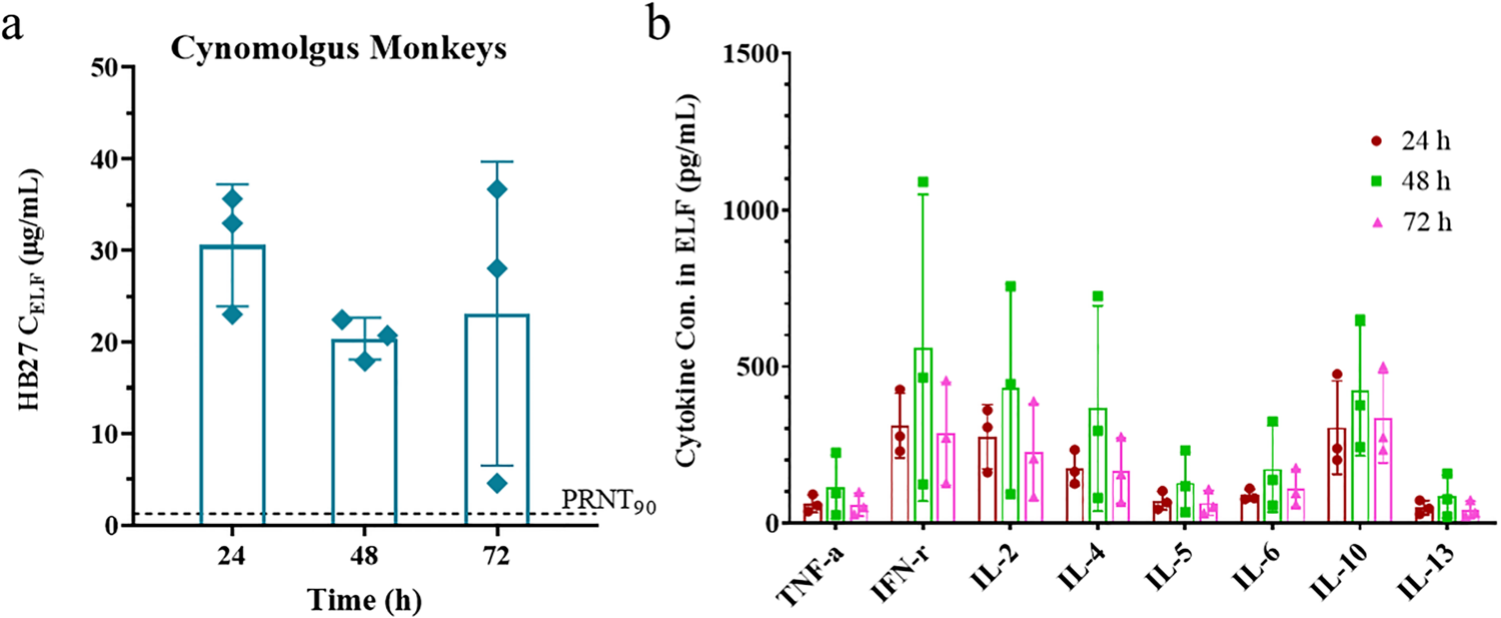
Lung PK and safety profile after single-dose inhalation of HB27 antibody in cynomolgus monkeys. The HB27 antibody concentrations (**a**) and cytokine levels (**b**) in ELF were measured at 24, 48 and 72 h after administration. Dashed line represents PRNT_90_ (1.28 μg/mL) of HB27 against authentic SARS-CoV-2. The data are shown as Mean ± SD. HB27 antibody in serum could not be quantified below the lower limit of detection (0.39 μg/mL).

The average antibody concentrations in ELF at 24 h, 48 h and 72 h were 30.56, 20.38 and 23.11 μg/mL, respectively. It is noticed that the antibody level in ELF had a slight decline from 24 to 72 h (Fig. 5a). The serum HB27 concentration was at or below the lower limit of detection (0.39 μg/mL, Table S4). The cytokine concentrations in ELF were also determined. No significant differences in cytokine concentration were observed among the three time points (P > 0.05) (Fig. 5b). Cytokine concentrations appeared to spike up at the 48 h time point, but all values were relatively low and recovered to normal levels at the 72 h time point. In addition, no abnormality was observed in the clinical observations and the macroscopic examination of organs in the respiratory tract.

## Discussion

Therapeutic neutralizing antibodies administered by intravenous infusion or intramuscular injection don’t transport efficiently through the plasma-lung barrier to inhibit viral infection in lung. Inhalation of nebulized antibody aerosol into lung could potentially reach a much higher local antibody concentration at a relatively low dose, and hence could achieve better antiviral efficacy and reduce dose to increase affordability of costly antibody treatments. HB27, a high-affinity neutralizing antibody against SARS-CoV-2, was shown to be potent and safe in preclinical studies and phase I trial via intravenous injection [1, 13]. The antibody Fc was modified with ‘LALA’ mutations to reduce antibody-dependent cellular cytotoxicity (ADCC) and antibody-dependent cellular phagocytosis (ADCP) functions without affecting its binding to FcRn. While recent studies for SARS-CoV-2 antibodies suggested that enhanced Fc-effector functions conferred better in-vivo protection [41–43]. The impacts of ‘LALA’ modification in HB27 on efficacy and safety need further investigation in clinical studies. Nevertheless, HB27 was chosen as a model antibody in this study to demonstrate the feasibility of delivering therapeutic antibodies to the respiratory tract including the lung via nebulized aerosol inhalation since a phase I study revealed its PK profile similar to a typical IgG1 antibody with T_1/2_ between 25.8 – 30 days in human [13].

Our C57BL/6 mice study confirmed that antibody concentrations in ELF were about 50-fold lower than those in serum when HB27 was injected through tail vein at 5 and 50 mg/kg doses, demonstrating that intravenous infusion is not an efficient method for antibody delivery to the lung for treating respiratory infectious diseases such as COVID-19, as the antibody concentration in ELF at 5 mg/kg dose was higher than the PRNT_50_ value but below the PRNT_90_ value of HB27 antibody. It should be noted that HB27 was shown to be highly potent against SARS-CoV-2 with extremely low PRNT_50_ and PRNT_90_ values of 32 ng/mL and 1.28 μg/mL respectively [1]. Furthermore, the in-vivo prophylactic and therapeutic activity of injected HB27 in hACE2 humanized mice showed a good efficacy with > 1000-fold reduction in lung viral levels [1]. When the delivery route was changed into the inhalation administration, the good PK profile of HB27 would maintain a higher concentration in the respiratory system and could potentially lead to further improvement in efficacy. Unfortunately, due to highly limited and stretched BSL-3 facilities/capacities as well as lack of experienced and trained personnel to conduct inhalation experiments under BSL-3 environment, we were unable to conduct an *in vivo* challenging study.

The mice inhalation studies demonstrated astonishing efficiency in delivering the antiviral antibody drug through nebulized aerosol administration to the target organ of viral infection. After a single dose of 5 mg/kg nebulized HB27 inhalation, the antibody concentration in ELF at 6 h was 857.82 μg/mL, 26,807-fold and 670-fold of the PRNT_50_ and PRNT_90_ values of HB27, respectively, and maintained above the PRNT_90_ value at 240 h. The peak inhaled HB27 concentration in ELF was 986-fold higher than the peak concentration in ELF (0.87 μg/mL) when administered with intravenous infusion at the same 5 mg/kg dose. In addition, the HB27 concentration in serum was also 2.0- to 5.9-fold of the PRNT_90_ value throughout the study period. SARS-CoV-2 infection typically lasts for a relatively short period of time, as majority of infected people recover within two weeks. Hence, a single dose of nebulized antibody inhalation could be sufficient to treat most COVID-19 patients.

The prominent PK characteristics of the antibody in ELF showed the T_1/2_ time of 44.4 h. As reported by Desoubeaux, inhaled macromolecules could be eliminated by alveolar macrophages and enzymes in the alveoli, and generally stay in the lungs for 1–2 days [30]. Our results showed that the antibody in ELF was not accumulated significantly after repeated intratracheal aerosol administrations of 5 mg/kg HB27 to the BALB/c mice, once daily for 5 times. Therefore, when the administration route is changed from intravenous injection to inhalation, a small amount of antibodies with multiple-dose could maintain a relatively stable antiviral drug concentration in the ELF to ensure the efficacy and avoid any possible safety risk of antibody accumulation in the lung.

One of the risks with aerosol inhalation administration is the potential toxicity caused by massive accumulation of macromolecules in the lungs causing immunogenic reaction [27]. HB27 is a humanized anti-SARS-CoV-2 antibody lacking ADCC or ADCP activity as a result of ‘LALA’ mutation in the Fc region, so it is unlikely to be immunogenic in human. Nevertheless, we measured the cytokine levels (including IL-6, TNF-α, IFN-γ, et al.) in ELF of cynomolgus monkeys after a single nebulized HB27 inhalation at about 4.64 mg/kg dose. No toxicological changes were noted at 24 h, 48 h and 72 h. Clinical observations and macroscopic examination of the respiratory tract also revealed no antibody-related abnormalities. In the repeat-dose study with intratracheal aerosol administration to BALB/c mice, the body weight and complete macroscopic examination after scheduled necropsy showed no abnormal changes as compared with the vehicle group. In consideration of the high concentration in ELF and reasonable T_1/2_ time, we expect only 1–2 doses of aerosol inhalation are needed for the treatment of COVID-19 in humans with no significant safety concerns.

## Conclusions

The pharmacokinetic characteristics and preliminary safety profile of HB27 antibody administrated through aerosol inhalation were investigated in mice and monkeys. Feasibility and significant advantages in efficient delivery of antiviral neutralizing antibody to the lung through aerosol inhalation were demonstrated for treatment of respiratory viral infection diseases such as COVID-19. These results support further preclinical development and future clinical applications.

## Supplementary Information

Below is the link to the electronic supplementary material.

Supplementary file1 (DOC 912 KB)

## Data Availability

Data supporting the findings of this study are available from the corresponding author upon reasonable request.

## Abbreviations

ADCC: Antibody-dependent cellular cytotoxicity
ADCP: Antibody-dependent cellular phagocytosis
ANR: Average nebulization rate
AR: Air flow rate
BALF: Broncho-alveolar lavages fluid
C_A_: Aerosol antibody concentration
C_BALF_: Concentration in BALF
C_ELF_: Concentration in ELF
C_P_: Formulation concentrations
C_max_: Maximum drug concentration
CHO: Chinese hamster ovary
COVID-19: Corona Virus Disease 2019
DLD: Delivered Lung Dose
ELF: Epithelial lining fluid
ELISA: Enzyme linked immunosorbent assay
EUA: Emergency Use Authorizations
FDA: Food and drug administration
HRP: Horse radish peroxidase
LD: Lung Dose
LDF: Lung deposition factor
LW: Lung weight
NCA: Non-compartmental model
PK: Pharmacokinetic
PRNT: Plaque reduction neutralization test
RBD: Receptor binding domain
RLU: Luminescence value
RMV: Respiratory minute volume
RT: Room temperature
SD: Standard deviation
SEC-HPLC: Size exclusion high-performance liquid chromatograph
T_1/2_: Half-life period
TDD: Total Delivered Dose
U_BALF_: Urea nitrogen concentration in BALF
U_ELF_: Urea nitrogen concentration in ELF
U_serum_: Urea nitrogen concentration in serum
WHO: World Health Organization
