# Supplementary material for: Feasibility Studies of Nebulized SARS-CoV-2 Neutralizing Antibody in Mice and Cynomolgus Monkeys
Source: Pharm Res. 2022 Jul 26;39(9):2191–201. doi: 10.1007/s11095-022-03340-9 (PMC9322739; doi:10.1007/s11095-022-03340-9)
Supplement: Supplementary file 1 — Supplementary file1 (DOC 912 KB) [file 11095_2022_3340_MOESM1_ESM.doc]

**Supplementary Materials: Feasibility Studies of Nebulized SARS-CoV-2 Neutralizing Antibody in Mice and Cynomolgus Monkeys**

Jilei Jia, Zhaojuan Yin, Xiao Zhang, Huimin Li, Dan Meng, Qianqian Liu, Hongfang Wang, Meng Han, Shixiang Suo, Yan Liu, Ping Hu, Chunyun Sun, Jing Li, and Liangzhi Xie

**Figure S1. Neutralizing activities of HB27 against SARS-CoV-2 pseudovirus of prevalent variants.** The dashed line represents the neutralization of antibody IC_50_ for prototype strain (8.0 ng/mL).

**Figure S2. Concentration-time profiles (Mean ± SD) in serum and ELF after intravenous administration (i.v.) of 50 mg/kg HB27 antibody in C57BL/6 mice (n=6).** The dashed line represents PRNT_90_ (1.28 μg/mL) of HB27 against authentic SARS-CoV-2.

**Table S1. Assessment of HB27 stability and binding activity upon nebulization by SEC-HPLC and ELISA**

| **HB27 antibody**  **Samples** | **Purity (SEC-HPLC, %)** | | | **Binding activity (ELISA)** | |
| --- | --- | --- | --- | --- | --- |
|  | Monomer | Aggregates | Fragment | Relative  activity | EC_50_ (ng/mL) |
| Prior to Nebulization | 99.6 | 0.4 | 0 | 102% | 2.1 |
| Post Nebulization | 99.7 | 0.3 | 0 | 118% | 1.8 |

**Table S2.** **HB27 antibody concentrations in serum and ELF after a single aerosol inhalation administration of 5 mg/kg HB27 to BALB/c mice (Mean ± SD,** μg/mL)

| **Time** | | **6 h** | **24 h** | **48 h** | **72 h** | **96 h** | **168 h** | **240 h** | **336 h** |
| --- | --- | --- | --- | --- | --- | --- | --- | --- | --- |
| **C_serum_**  **(μg/mL)** | Mean | 2.56 | 4.24 | 5.61 | 5.80 | 4.97 | 5.61 | 7.59 | 6.56 |
|  | SD | 1.22 | 0.80 | 2.06 | 1.63 | 0.66 | 1.45 | 4.04 | 1.84 |
| **C_ELF_**  **(μg/mL)** | Mean | 857.82 | 560.42 | 245.43 | 115.93 | 71.80 | 8.94 | 2.37 | 0.64 |
|  | SD | 117.67 | 111.87 | 30.34 | 29.58 | 24.75 | 2.30 | 0.83 | 0.64 |

**Table S3. The minute ventilation and aerosol concentration of 3 jet-type NE-C28 nebulizers used in the inhalation study with cynomolgus monkeys**

| **Nebulizer ID** | **AR**  **(mL/min)** | **T**  **(min)** | **DV**  **(mL)** | **ANR**  **(mL/min)** | **C_P_**  **(mg/mL)** | **C_A_**  **(mg/mL)** |
| --- | --- | --- | --- | --- | --- | --- |
| Nebulizer 1 | 4971.36 | 4 | 1.2 | **0.23±0.07** | 10 | **0.00045** |
|  |  | 6 | 1.3 |  |  |  |
|  |  | 4 | 1.0 |  |  |  |
|  |  | 6 | 0.8 |  |  |  |
| Nebulizer 2 | 4730.94 | 4 | 1.1 | **0.19±0.06** | 10 | **0.00041** |
|  |  | 6 | 1.0 |  |  |  |
|  |  | 4 | 0.6 |  |  |  |
|  |  | 6 | 1.0 |  |  |  |
| Nebulizer 3 | 5995.74 | 4 | 1.2 | **0.24±0.05** | 10 | **0.00039** |
|  |  | 6 | 1.3 |  |  |  |
|  |  | 4 | 1.0 |  |  |  |
|  |  | 6 | 1.0 |  |  |  |

Notes: AR (mL/min) means the air flow rate of the nebulizer, T (min) is inhalation exposure time, DV is the dosing volume of HB27 liquid formulation, ANR (mL/min) is the average nebulization rate of the nebulizer, C_P_ (mg/mL) is the concentration of HB27 liquid formulation, and C_A_ (mg/mL) is the concentration of aerosol from nebulizer. ANR = DV / T, C_A_ = ANR × C_P_ / AR.

**Table S4. HB27 antibody concentrations in serum after** single aerosol inhalation in cynomolgus monkeys

| **Group** | **Animal ID** | **TDD**  **(mg/kg)** | **Antibody Con. (μg/mL) –Time point** | | | | | |
| --- | --- | --- | --- | --- | --- | --- | --- | --- |
|  |  |  | 0 h | 1 h | 6 h | 24 h | 48 h | 72 h |
| **24 h** | 1# | 4.25 | ND | ND | ND | ND | - | - |
|  | 5# | 3.86 | 0.48 | 0.48 | 0.52 | 0.66 | - | - |
|  | 6# | 3.64 | ND | ND | ND | ND | - | - |
| **48 h** | 2# | 2.76 | ND | ND | ND | ND | ND | - |
|  | 3# | 2.28 | ND | ND | ND | ND | ND | - |
|  | 7# | 10.61 | ND | ND | ND | ND | ND | - |
| **72 h** | 4# | 2.74 | ND | ND | ND | ND | ND | ND |
|  | 8# | 5.78 | ND | ND | ND | ND | ND | ND |
|  | 9# | 5.83 | ND | ND | ND | 0.50 | 0.57 | 0.55 |

Notes: “ND” means no detectable, which the data is below the lower limit of detection (0.39 μg/mL); “-” is that the monkey had been euthanized and no serum sample was obtained.
